# Characterization of the Akt2 Domain Essential for Binding Nuclear p21cip1 to Promote Cell Cycle Arrest during Myogenic Differentiation

**DOI:** 10.1371/journal.pone.0076987

**Published:** 2013-10-23

**Authors:** Lisa Heron-Milhavet, Celine Franckhauser, Anne Fernandez, Ned J. Lamb

**Affiliations:** Cell Cycle and Myogenesis, Institute of Human Genetics, CNRS-UPR1142, Montpellier, France; McGill University, Canada

## Abstract

The binding of the cdk inhibitor p21cip1 to Akt2 in the nucleus is an essential component in determining the specific role of Akt2 in the cell cycle arrest that precedes myogenic differentiation. Here, through a combination of biochemical and cell biology approaches, we have addressed the molecular basis of this binding. Using amino-terminal truncation of Akt2, we show that p21cip1 binds at the carboxy terminal of Akt2 since deletion of the first 400 amino acids did not affect the interaction between Akt2 and p21cip1. Pull down using carboxy terminal-truncated Akt2 protein revealed the importance of the region between amino acids 400 and 445 for the binding to p21cip1. Since Akt2_400–445 and Akt2_420–445 peptides could both bind p21cip1, this refines the binding domain on Akt2 between amino acids 420 and 445. In order to confirm these data in living cells, we developed a protocol to synchronize myoblasts at the cell cycle exit point when p21cip1 expression is induced by MyoD before myogenic differentiation. When a synthetic Akt2 peptide spanning the region (410–437) was microinjected in p21-expressing myoblasts, p21cip1 no longer localized exclusively in the nucleus, instead being redistributed throughout the cell, thus showing that injected peptide 410–437 acts to compete with the binding of endogenous Akt2 to p21cip1. Taken together, our data suggest that this 27 amino acid sequence on Akt2 is necessary and sufficient to bind p21cip1 both *in vitro* and in living cells.

## Introduction

The serine-threonine kinase Akt was first discovered as the oncogene in the transforming retrovirus AKT8 [1] and it has become the subject of intense research since then because of its implication in cancer progression, metabolism, cellular growth and differentiation, and survival. Three isoforms of Akt have been identified: Akt1, Akt2 and Akt3 and their tissue distribution has been determined [2] showing that both Akt1 and Akt2 isoforms are ubiquitously expressed, whereas the Akt3 isoform is not detected in several tissues where Akt1 and Akt2 are highly expressed, but is relatively highly expressed in brain and in testis. Akt2 is expressed predominantly in insulin target tissues, such as fat cells, liver and skeletal muscle. The three Akt isoforms possess the kinase domain in the central region of the molecule; the PH (pleckstrin homology) domain acts as phosphoinositide-binding molecule and the hydrophobic motif (HM) is located at the carboxy-terminal adjacent to the kinase domain [3]. Akt is activated by a multistep process that results in phosphorylation of two critical residues threonine 308 in the activation loop and serine 473 in the hydrophobic motif, which induces a substantial conformational change that leads to a greater than 1000-fold increase in its kinase activity [4]–[6]. The initiation step in the activation of Akt is its recruitment to the plasma membrane where the PH domain directs the translocation of Akt from the cytosol to the plasma membrane by binding to the products of PI3K. We have published in 2006 and in 2008 the respective role of Akt1 and Akt2 isoforms in the regulation of cell cycle proliferation and exit towards myogenic differentiation. We have shown that Akt1 is implicated in cell cycle progression whereas Akt2, principally through its interaction with the cdk inhibitor p21cip1, is implicated in cell cycle exit thus promoting myoblast differentiation [7]–[8].

In this study, we have determined the region on Akt2 necessary for the binding with p21cip1 is a 27 amino acid sequence spanning the C-terminal region 410–437 of Akt2, showing strong differences with Akt1 in both primary sequence and secondary structure.

## Results and Discussion

We have previously shown that Akt2 interacts with p21cip1, inducing its stabilization in the nucleus promoting cell cycle arrest and entry into myogenic differentiation [7]. In the present study, we have cloned, produced and purified various forms of human Akt2 proteins truncated specifically at the N-terminal and C-terminal Akt2 regions (see Figure S1 for details) as well as Akt2-derived peptides to study the binding site between Akt2 and p21.

**Figure 1:**
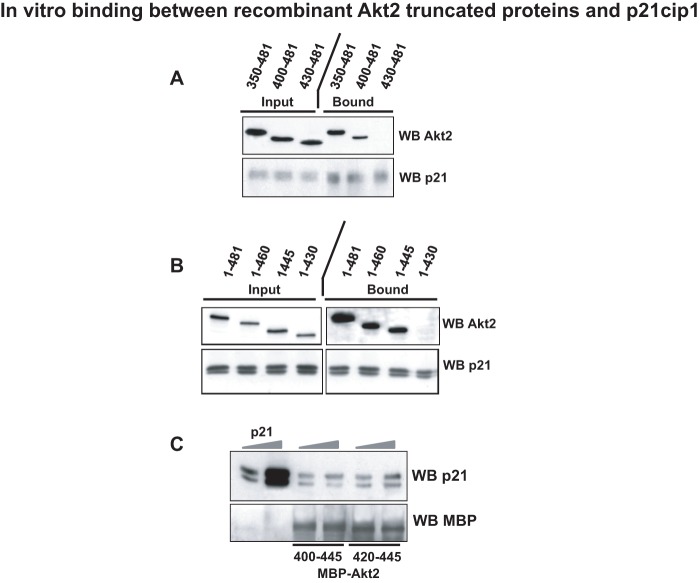
In vitro binding between recombinant human Akt2 truncated proteins and human p21cip1. A. Pull-down assay was performed between each of three recombinant N-terminal truncated Akt2 proteins (350–481, 400–481, 430–481) and recombinant human p21cip1. Inputs are shown on the left and pull down assays on the right. Membranes were blotted with Akt2 and with p21 as a control. B. Immunoprecipitations (*in vitro*) were performed between each of four recombinant full length Akt2 (1–481), C-terminal truncated Akt2 proteins (1–460, 1–445 and 1–430) and human recombinant p21cip1. Inputs are shown on the left and pull-down assays on the right. Membranes were blotted with Akt2 and with p21 as a control. C. Pull-down assay was performed between Akt2 peptides (400–445) or (420–445) and recombinant p21cip1. Increasing inputs of p21 (1 µg and 5 µg) are shown on the left lanes and pull-down assays with increasing amounts of p21 on the right lanes. Membranes were blotted with p21 and with MBP as a control.

We first examined whether p21cip1 bound to Akt2 through its amino or carboxy terminal region. Three N-terminal deletions of Akt2 were expressed in bacteria with C-terminal 6 His fusion tag and after purification, these deletion proteins were probed for the capacity to bind p21 by pull down assay using anti-p21. As shown in figure 1A, deletion of amino acids 1–350 or 1–400 did not affect the capacity of Akt2 to bind p21. In contrast, a truncated form of Akt2 in which only the amino acids 430–481 remained was unable to bind p21 *in vitro*. To confirm the importance of the C-terminal region of Akt2 in the binding to p21, we next performed the reciprocal experiment, truncating regions from the C-terminus. As shown in figure 1B, truncation of amino acids 460–481 and 445–481 had no effect on the interaction with p21 whereas a truncation spanning amino acids 430–481 was no longer capable of binding p21. These data suggest that a region between amino acids 400–445 of Akt2 is necessary for the binding to p21 *in vitro*. This region corresponds to the site at which the primary sequences of Akt1 and Akt2 diverge the most, consistent with being potentially the sequence through which Akt2 binds specifically p21cip1, an interaction never observed with Akt1 [7]. We next examined if we could reduce this region by producing Akt2 peptides as maltose binding fusion proteins (MBP). This has the dual advantage that the interactions observed above occur through the presence of 6 Hi sequences in both p21 and Akt2 truncations and that the complexes formed can be extensively washed to reinforce the robustness of the binding. As shown in figure 1C, when p21cip1 was incubated with either Akt2 (400–445) or Akt2 (420–445), there was a clear binding to both truncation peptides. The binding between Akt2 (400–445) and p21cip1 was even slightly less efficient than that of Akt2 (420–445) since it showed no increase in p21 binding when the levels of p21 were increased by 5 times. In contrast, the peptide Akt2 (420–445) showed a modest increase in the levels of p21 retained, although not in direct correspondence with the increase in p21 load. Together, these data would suggest that the region between amino acids 420 and 430 on human Akt2 is sufficient for the binding to p21cip1.

**Figure 2:**
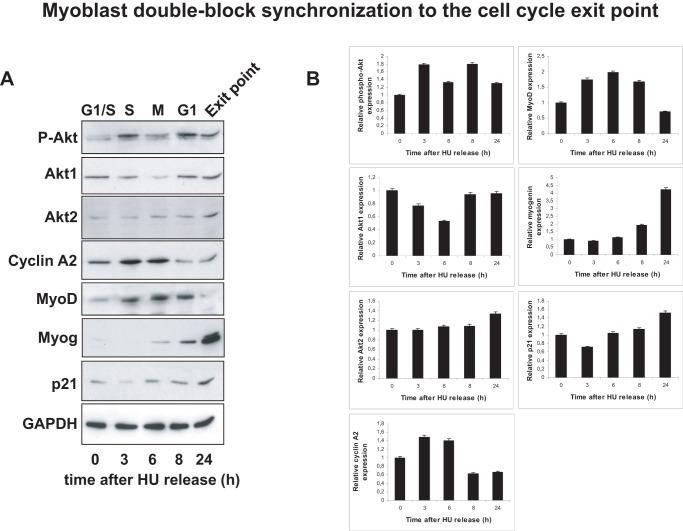
Myoblast double-block synchronization to the cell cycle exit point – Part 1. A. Total proteins were extracted from synchronized myoblasts 3, 6, 8 and 24-block release. 20 µg of total proteins were loaded onto SDS-PAGE gels and blotted for phospho-Akt (P-Akt), Akt1, Akt2, cyclin A2, MyoD, myogenin, p21cip1 and GAPDH as a loading control. B. Quantification of phospho-Akt, Akt1, Akt2, cyclin A2, MyoD, myogenin and p21cip1 blots as a relative expression as compared to GAPDH protein expression (using Image J software). This experiment has been reproduced 4 times and Y error bars are shown on the graphs.

**Figure 3:**
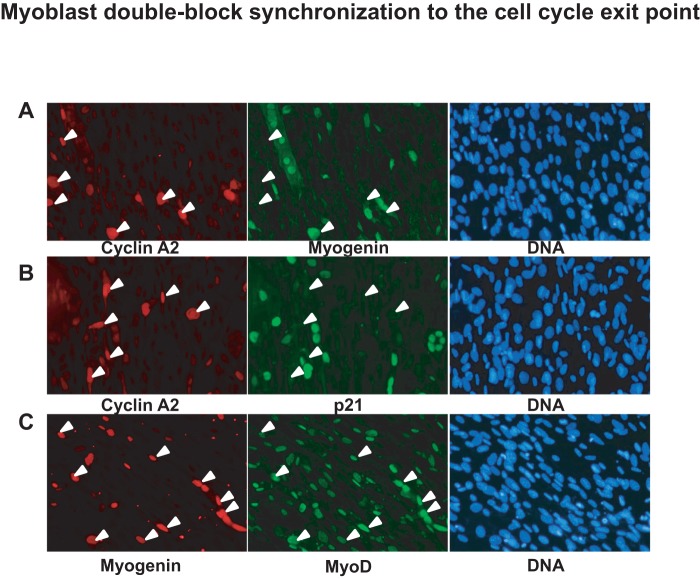
Myoblast double-block synchronization to the cell cycle exit point – Part 2. C2.7.4 myoblasts were fixed 24-block release. Immunofluorescence was performed revealing either cyclin A2 with myogenin (A), cyclin A2 with p21 (B) and myogenin with MyoD (C). Secondary Alexa-fluor-488 and −455 antibodies were used for immunofluorescence detection and DNA was labeled with Hoechst. White arrows shows cyclin A2 positive cells (A and B) and myogenin positive cells (C).

In order to confirm these data in living cells, we needed a cell system in which endogenous p21cip1 levels would increase in a manageable manner. During myoblast differentiation, cells first exit the cell division cycle before the induction of full myogenesis, a time point corresponding to a reproducible increase in endogenous p21cip1 expression. We therefore developed a protocol to synchronize myoblasts at the cell cycle exit point using a combination of serum and amino acid deprivation. Without any synchronization, less than 20% of the myoblasts reach the cell cycle exit point simultaneously. Based on the protocol previously established by our group [9], we have performed double-block synchronization. The first block to a G0 like state involved a 48 h methionine-free starvation followed by a brief refeed into proliferation (high serum) immediately followed by the second block at G1/S by a 15 h hydroxyurea (HU) incubation (see Methods for details). Finally, the HU block was released using differentiation medium containing 2% FBS. As described before [9], methionine deprivation blocks myoblast proliferation without induction of differentiation and allows the myoblasts to be in a quiescent state (G0). In order to identify conditions in which the second block was accompanied by the highest levels of p21cip1 expression, cells were released into HU-containing low serum at different levels of confluence and the protein expression profiles examined at different times after HU release. Total proteins obtained after HU-block release were firstly separated by SDS-PAGE and membranes were then blotted with different antibodies to analyze proliferation, cell cycle arrest and exit to myogenic differentiation. As shown in figure 2A (quantified in figure 2B), myoblasts reached S phase 3 to 6 hours after HU release, as indicated by cyclin A2 expression, and attained mitosis (M phase) 6 to 8 hours after HU release as shown by MyoD expression and consistent with our previous findings [9]. Moreover, the cell cycle exit point was reached 24 hours after HU release as indicated by myogenin expression accompanied by p21cip1 and an increased expression of Akt2, reaching a maximum 24 hours after HU release [8]. Interestingly, we have analyzed the expression and activity (phospho-Akt) of Akt1 during this synchronization and observed that the expression of active Akt was increased during S phase and decreased during mitosis while Akt1 protein was still expressed at the G1/S boundary (figure 2). We next performed immunofluorescence 24 hours after the HU-block release. Proliferation was followed by cyclin A2 expression and cell cycle exit into myogenic differentiation was confirmed by the expression of myogenin in myocytes and myotubes (figure 3A). p21cip1, as a cdk inhibitor, is also expressed in the nucleus of cells that are no longer cycling *i.e.* cyclin A2 negative cells (figure 3B). We have also noticed that maximal levels of MyoD were present in cells that are ready to exit the cell cycle and to enter myogenic differentiation since they express myogenin (figure 3C). These data show that myoblasts can be reliably synchronized at the cell cycle exit point, expressing both MyoD and p21cip1 and that effective cell cycle exit requires the cells to be confluent when released from the amino acid deprivation.

**Figure 4:**
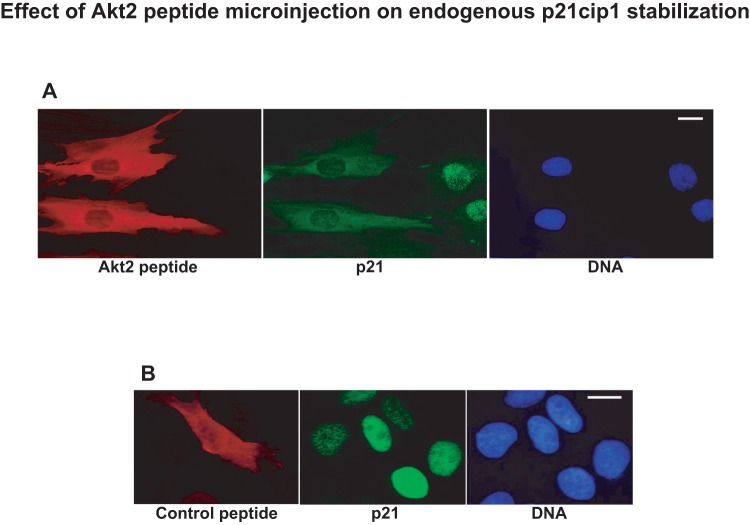
Effect of Akt2 peptide microinjection on endogenous p21cip1 protein stabilization. The Akt2 synthetic peptide (410–437) or a control peptide (spanning amino acids 425–452 of cdc25C) were microinjected into quiescent human myoblasts and cells were fixed 40 minutes after to avoid peptide degradation before immunofluorescence to detect endogenous p21cip1 and check for its localization/stabilization. Bars in both panels  = 10 µM.

Taking advantage of this newly established protocol of myoblast synchronization, we next examined the effect a synthetic peptide corresponding to the region 410–437 of Akt2. The choice of this peptide was based on the maximum divergence with the sequence in Akt1. We microinjected the Akt2 peptide spanning amino-acids 410–437 into quiescent human myoblasts and checked for its effect on p21cip1 protein stabilization at different times afterwards. Figure 4 shows human myoblasts fixed 40 minutes after peptide microinjection and stained for p21. Cells microinjected with Akt2 (410–437) showed no longer p21 nuclear localization. In contrast, cells injected with a control peptide corresponding to amino acids 425–452 of human cdc25C showed no effect on the localization of p21. Interestingly, in cells injected with Akt2 (410–437) there was frequently a close correspondence between the diffuse cytoplasmic localization of the peptide and the staining for p21. In cells microinjected with the Akt2 peptide and incubated for longer periods (100 min), the localization of p21 returned to normal suggesting that the peptide may have been degraded which is consistent with our previous observation that peptides containing frequent lysine or arginine residues have a short half life in vitro [10]. We believe that the (410–437)-Akt2 peptide competes with endogenous Akt2 for the binding to p21cip1, thus displacing it from the nucleus where Akt2 is localized.

Taken together, these data show that a short amino acid region from 400 to 445 was sufficient to bind p21cip1. More specific analysis by microinjection in living cells revealed that a peptide covering the region 410 to 437 of Akt2 was sufficient to induce the delocalization of p21 from the nucleus. This shorter sequence corresponds directly to the region of maximum dissimilarity between Akt1 and Akt2 which strengthen the likelihood that a sequence around this region would be responsible for p21 binding.

**Figure 5:**
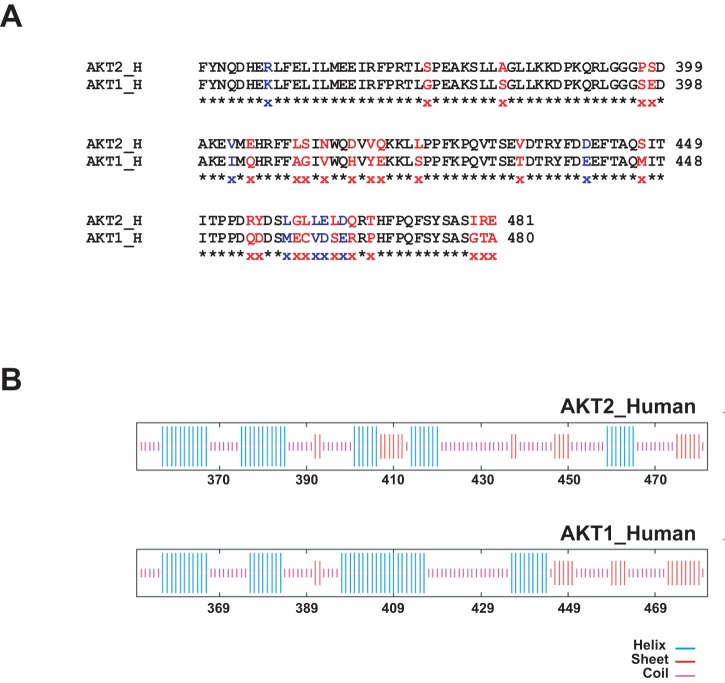
Sequence homology and secondary structure prediction of human Akt1 and Akt2. A. Sequence alignment for the last 130 amino acids of human Akt1 and Akt2. Different residues are shown in red and similar residues are shown in blue. B. Secondary structure prediction for the last 130 amino acids of human Akt1 and Akt2 using GOR4 shared software from PBIL, France (http://npsa-pbil.ibcp.fr/cgi-bin/npsa_automat.pl?page=npsa_gor4.html).

As shown Figure 5A and 5B, and previously discussed [11], the region around amino acid 410 is predicted to be essentially alpha-helix in Akt1 whereas it is mostly beta-sheet in Akt2. Similarly, whereas the sequence between amino acids 415 and 437 is also predicted to be coiled-coil in both Akt1 and Akt2, there is a sequence around amino acid 435 in Akt2 that is beta-sheet. Interestingly at the amino acid level, major differences between Akt1 and Akt2 exist between amino acids 410 and 424 (Figure 5A). These differences include substitution of acidic amino acids for hydrophobic amino acids (valine for tyrosine at position 418) of glutamine for glutamic acid at position 419) or substitution of serine to leucine at position 424. The latter is potentially important since serine 424 is followed by a proline and may correspond to a putative cdk motif present on Akt1 and absent from Akt2.

Interestingly, our data using truncated forms of Akt2 are also consistent with previous predictions from structural analysis of Akt/PKB [6], [12]. In the latter, Barford and colleagues predicted that the phosphorylated hydrophobic motif of Akt (470–475) folded back to the C-terminal region of the kinase to interact with the kinase domain surrounding threonine 309 inducing a disorder to ordered transition associated with Akt activation. As a result of this fold back transition, aspartic acid 440 was predicted to directly influence substrate specificity of both Akt isoforms through a potential interaction with arginine groups (at position −3) in Akt substrates. By binding to the region 410–437, p21cip1 would thus hamper the possibility for Akt2 to become activated, which may well explain why p21cip1 while having an effective Akt phosphorylation motif (which is phosphorylated by Akt1), was not phosphorylated by Akt2. It will now be interesting to determine if antibodies to the (410–437)-peptide are effective inhibitors of Akt2. If this turns out to be the case, both Akt2 (410–437) and antibodies against this region would represent powerful tools for identifying Akt2-dependent events in cell transformation.

## Materials and Methods

### Cell culture

C2.7.4 mouse myoblasts were grown in proliferation medium: Dulbecco's Modified Eagle's Medium HAM F12 medium (DMEM HAM F12, Sigma) supplemented with 10% fetal calf serum (FCS, Eurobio), penicillin/streptomycin (100 µg/ml) and glutamine (2 mM). To induce differentiation, C2 myoblasts were cultured in differentiation medium containing DMEM HAM F12, 2% FCS, antibiotics and glutamine. Human primary myoblasts were obtained from a muscle needle biopsy and were grown in the same medium as C2.7.4 myoblasts [8].

### Cloning, production and purification of His-tagged C terminal truncated Akt2 proteins

C-terminal truncated Akt2 mutants have been amplified by PCR from the pBluescript II-Akt2 plasmid, sub-cloned into pET101/D-TOPO and all have been expressed and purified using the Champion pET Directional TOPO Expression Kit according to the manufacturer's recommendations (Invitrogen). Recombinant polyhistidine-tagged proteins were purified using the TALON Metal Affinity Resin (Clontech) under native conditions and according to the manufacturer conditions. Akt2 (1–481), Akt2 (1–460), Akt2 (1–445) and Akt2 (1–430) truncated and tagged proteins were used for the binding study with p21cip1.

### Cloning, production and purification of MBP-tagged N terminal truncated Akt2 proteins

N-terminal truncated Akt2 mutants were sub-cloned into pMAL-p4X expression vector and mutants were expressed and purified using the pMAL Protein Fusion and Purification System (NEB). Recombinant MBP-tagged proteins were purified by Amylose affinity chromatography resin and eluted with Maltose (10 mm) under native conditions and according to the manufacturer instructions.

Truncated Akt2 (350–481), Akt2 (400–481) and Akt2 (430–481) proteins were used for the binding studies with p21cip1.

### Akt2 peptide

The following peptide spanning amino acids 410 to 437 in the Akt2 sequence has been synthesized.

FLSINWQVVQKKLLPPFKPQVTSEVDT (Genecust, Luxembourg) and was used directly diluted at 1 mg/ml for microinjection experiments. As a control, cells were microinjected corresponding to amino acids 425 to 452 of human cdc25C.

### *In vitro* pull-down assay

Pull-down assays [7] using Akt2 truncated proteins and recombinant p21cip1 have been performed by one of two methods: When both Akt2 mutant proteins and p21cip1 were His-tagged, purified proteins were incubated together on ice before immunoprecipitation of p21cip1 and probing the immunoprecipitates of p21 for Akt2 by Western blot. Alternatively, when Akt2 proteins were MBP-tagged, Akt2 proteins and peptides were bound to the amylose resin. After incubation with p21, amylose resin was extensively washed and maltose-fusion peptides were eluted, separated by SDS-PAGE before blotting for the presence of p21 with anti-p21 antibody.

### Myoblast double block synchronization at the cell cycle exit point

In order to obtain cell expressing p21, we developed a protocol to synchronize mouse or human myoblasts at the cell cycle exit point. Myoblasts were plated at different confluence on 35 mm dishes for immunoprecipitations and western blotting, or on glass coverslips for immunofluorescence. 24 hours after plating, cells were rinsed twice in PBS and then shifted in DMEM without methionine for 48 hours. Quiescent (G0) myoblasts were allowed to re-enter the cell cycle by changing the medium first to fresh DMEM HAM F12 containing 10% foetal bovine serum as a jump start for 2 hours. Finally cells were synchronized at the G1/S boundary by adding 0.1 mM hydroxyurea (HU) in differentiation medium (DMEM HAM F12 supplemented with 2% foetal bovine serum) 2 hours after release of methionine deprivation and for a total period of 15 hours. Cells were then extensively washed sequentially in PBS for 30 sec, PBS for 5 min and finally in DMEM HAM F12 without serum for 15 min. Cells were then cultured back into differentiation medium for 0, 3, 6, 8 and 24 hours after HU wash. Maximal S phase, M phase and the cell cycle exit point were obtained 2–3, 6–7 and 24 hours respectively after release of HU. To determine S phase entry, 0.1 mM 5-bromodeoxyuridine (BrdU) was added to cells during 15 min after HU release. This protocol was adapted from Kitzmann et al, 1998 and modified in order to explore the cell cycle exit point before entry of post-mitotically arrested myoblasts into myogenic differentiation [9].

### Immunofluorescence

Immunofluorescence experiments were conducted as previously described [7]. Briefly, cells were fixed in formalin and extracted with cold acetone before incubation with monoclonal anti-myogenin F5D (BD biosciences), polyclonal rabbit anti-p21 and rabbit anti-MyoD (Santa Cruz, Belgium) and rabbit anti-cyclin A2 primary antibodies [13]. The specific staining was revealed using anti-mouse Alexa Fluor 488- and anti-rabbit Alexa Fluor 555-labeled secondary antibodies (Invitrogen).

### Western blot analysis

Protein extracts from C2.7.4 mouse myoblasts were prepared in protein extraction buffer containing SDS 1%, Tris HCl 40 mM pH 6,8 and glycerol 7,5%. Fifteen to 20 µg of total protein were loaded in reducing buffer and separated on a 10% SDS/PAGE gel. Resolved proteins were transferred to nitrocellulose and incubated rabbit anti-p21 or anti-myogenin (Santa Cruz), anti-MBP (NEB), anti-Akt2, anti-Akt1 or anti phospho S473 Akt (Cell Signaling Technology) Secondary antibodies were horseradish peroxidase-conjugated anti-rabbit or anti-mouse antibodies (Jackson Immunoresearch Lab., Charles River, France). All blots were developed by using an ECL chemiluminescence reagent (Lumilight, Roche).

## Supporting Information

Figure S1 **Schematic representing the Akt2 truncated proteins.** This figure represents a schematic view of Akt2 full-length (1–481) as well as N-terminal and C-terminal Akt2 truncated proteins used for immunoprecipitation, pull down and cell transfection experiments.
(EPS)
